# Optimization of Conditions for a Freeze-Dried Restructured Strawberry Block by Adding Guar Gum, Pectin and Gelatin

**DOI:** 10.3390/plants11212809

**Published:** 2022-10-22

**Authors:** Jiaqi Hu, Xiyun Sun, Hongwei Xiao, Feifei Yang, Chunju Liu, Haiou Wang, Honglin Zhang, Wei Zhang

**Affiliations:** 1 College of Food Science, Shenyang Agricultural University, Shenyang 100866, China; 2 School of Food Science, Nanjing Xiaozhuang University, Nanjing 211171, China; 3 College of Engineering, China Agricultural University, Beijing 100083, China; 4 Institute of Agro-Product Processing, Jiangsu Academy of Agricultural Sciences, Nanjing 210014, China

**Keywords:** freeze-dried restructured strawberry block, edible gum, textural property, quality, response surface methodology

## Abstract

With its high moisture content and tender texture, fresh strawberry is very susceptible to mechanical damage and microbial infection. Drying is one of the most frequently employed methods to extend its shelf life, and freeze-dried restructured strawberry block (FRSB) is an emerging popular food. Here, in order to enhance the quality of FRSB, edible gums of guar gum, pectin, and gelatin were added and the combination was optimized using response surface methodology (RSM) with chewiness, hardness, and organoleptic evaluations of the dried sample as the response indicators. The results showed that the combination addition of 0.10% guar gum, 0.22% pectin, and 0.30% gelatin contributed to the highest comprehensive quality of the dried sample. Compared with the untreated sample, the optimal combination addition of the three edible gums resulted in a higher moisture content for the dried sample (increased by 0.8%), and increased the chewiness, hardness, and porosity by 82.04%, 27.09%, and 3.01%, respectively, while maintaining more original color and forming a denser porous microstructure. The findings in the current work will be useful for the application of edible gums in freeze-dried restructured fruits and vegetables.

## 1. Introduction

Strawberry (*Fragaria ananassa Duch.*) is a popular fruit due to its unique taste and rich nutritional value [1,2]. According to the literature data, strawberries also have anticancer, anti-inflammatory, and antidiabetic functions [3,4,5]. However, strawberries have a short maturity period, are very perishable, and are extremely prone to quality loss due to mechanical damage or improper storage after harvesting. Therefore, to prolong their shelf life, it is important to process and preserve strawberries after harvesting [6,7]. Vacuum freeze-drying has been proven to be a preferred drying method to preserve strawberries, contributing to a better quality in dried strawberry puree and strawberry slices [8,9].

Recently, freeze-dried restructured fruit and vegetable products (for example, restructured rose flower [10], yam [11], and wild cabbage [12]) have gradually emerged in the market and are favored by consumers. Freeze-dried restructured fruit and vegetable products have several advantages over natural fruit and vegetable chips and slices [13]. The freeze-dried restructured food could be used for personalized foods for different groups of people with different needs by reasonably combining different fruits, vegetables, and raw food materials in proportion to meet the needs of different consumers [14]. The formation of a uniform pulp can avoid adverse effects on the sublimation of water due to epidermal wax and tissue cell barriers during the freeze-drying process, thereby improving the drying rate and quality [15]. Currently, to the best of our knowledge, no effort has been devoted to the development of freeze-dried restructured of strawberry products.

Edible gum is a unique food additive that is widely used in a variety of foods in daily life. It has many important functions, such as thickening, gelling, emulsifying, stabilizing, and clarifying, and is often used in frozen foods, beverages, dairy products, candy, and the refrigerated food industry [16,17]. Studies have shown that edible gums can improve the quality of foods. It was found that the gum arabic (*Acacia senegal*) edible coating improved the quality and postharvest-shelf life of cold-stored strawberry fruit [18]. Lin et al. employed xanthan gum and konjac gum to improve the viscoelasticity of mung bean resistant starch, significantly enhancing the K value and dynamic moduli with increasing the gum concentration [19]. However, to the best of our knowledge, no reports have been found detailing the effect of edible gum addition on drying behavior and quality of FRSB.

Guar gum, a dietary fiber [20]; pectin, an important polysaccharide [21]; and gelatin, a protein obtained by the hydrolysis of collagen [22], all act as stabilizers in food. Pre-experimental research found that each addition of guar gum, pectin, and gelatin all contributed to a better comprehensive quality in the FRSB. However, the combination effects of the three edible gums on the FRSB are still unclear. Three kinds of edible gums from different sources were selected and added to FRSB as the research focus of this paper. The purpose of this study was to determine the optimal amount of compound edible gums added to the FRSB to obtain the best comprehensive quality and to compare the quality of the optimized FRSB with that of the FRSB without edible gum. These findings will help the application of edible gum in the freeze-dried restructured fruits and vegetables and provide a reference for the expansion of fresh strawberry sales and the development of the pulp processing industry.

## 2. Results and Discussion

### 2.1. Analysis of Variance of the Regression Models

Food texture characteristics are a set of physical properties derived from the structure of the food, and the texture profile analysis (TPA) and puncture test were performed on the products to evaluate their texture properties. In the TPA test, the samples were compressed twice to simulate the chewing effect of teeth so as to evaluate their textural properties, and the chewiness index was used for TPA evaluation, reflecting the energy required by the teeth to chew FRSB into a swallowing state, and comprehensively reflecting the continuous resistance of the samples to chewing. In the puncture test, the hardness index was used for puncture evaluation. The organoleptic evaluation indicators reflect the consumer’s acceptance of the product, including crispness, stickiness, acidity, and sweetness. Analysis of variance (ANOVA) of the regression model was performed using Design Expert 8.0 software to study the statistical significance of the independent variables of guar gum (A), pectin (B), and gelatin (C) for chewiness (Y_1_), hardness (Y_2_), and organoleptic evaluations (Y_3_). The results are presented in Table 1, with the data fitted using quadratic multiple regression, and the obtained quadratic regression equation for chewiness, hardness, and organoleptic evaluation are shown in Equations (1–3), respectively.

(1) $$Y_{1}=16.17\;-\;8.05\;\times \;A\;-\;9.08\;\times \;B+2.46\;\times \;C+9.14\;\times \;A\;\times \;B\;-\;8.09\;\times \;A\;\times \;C+2.95\;\times \;B\;\times \;C+1{.97\;\times \;A}^{2}+3{.83\;\times \;B}^{2}+3{.02\;\times \;C}^{2}$$

(2) $$Y_{2}=28.97\;-\;2.35\;\times \;A+2.94\;\times \;B\;-\;0.34\;\times \;C\;-\;0.48\;\times \;A\;\times \;B+0.77\;\times \;A\;\times \;C+0.27\;\times \;B\;\times \;C\;-\;1.85\;\times \;A^{2}\;-\;8.65\;\times \;B^{2}\;-\;1.92\;\times \;C^{2}$$

(3) $$Y_{3}=8.19\;-\;0.12\;\times \;A+0.23\;\times \;B\;-\;0.029\;\times \;C\;-\;0.17\;\times \;A\;\times \;B+0.020\;\times \;A\;\times \;C+0.092\;\times \;B\;\times \;C\;-\;0{.27\;\times \;A}^{2}\;-\;0{.25\;\times \;B}^{2}\;-\;0{.052\;\times \;C}^{2}$$

As shown in Table 1, the quadratic regression models of the chewiness, hardness, and organoleptic evaluation index were all significant (*p* < 0.05), the *F*-values were 5.51, 6.66, and 5.19, respectively, in the three models; lack of fit were not all significant (*p* > 0.05) and the *F*-values were 3.09, 1.67, and 1.20, respectively, for the models, indicating that the models fit well with the experimental data, thereby validating the models. Therefore, the models can be used to predict the effect of edible gum addition on the chewiness, hardness, and organoleptic evaluation of the FRSB. In the quadratic regression model, A, B, AB, and AC had significant effects on the chewiness evaluation index, B and B^2^ had significant effects on the hardness index, and B, A^2^ and B^2^ had significant effects on the sensory evaluation index (*p* < 0.05). The results of the sum of square showed that the effect of the three factors on the chewiness, hardness, and organoleptic evaluation was in the order of pectin > guar gum > gelatin. This may be because that pectin exhibits good adhesiveness behaviors, and its branched arabinose and galactose are easily cross-linked with cell matrix components including hemicellulose, cellulose, and others in the strawberry tissues, which is more conducive to form a rigid network structure [23].

### 2.2. Influence of the Amount of Edible Gum Added on the Chewiness Evaluation

**Figure 1:**
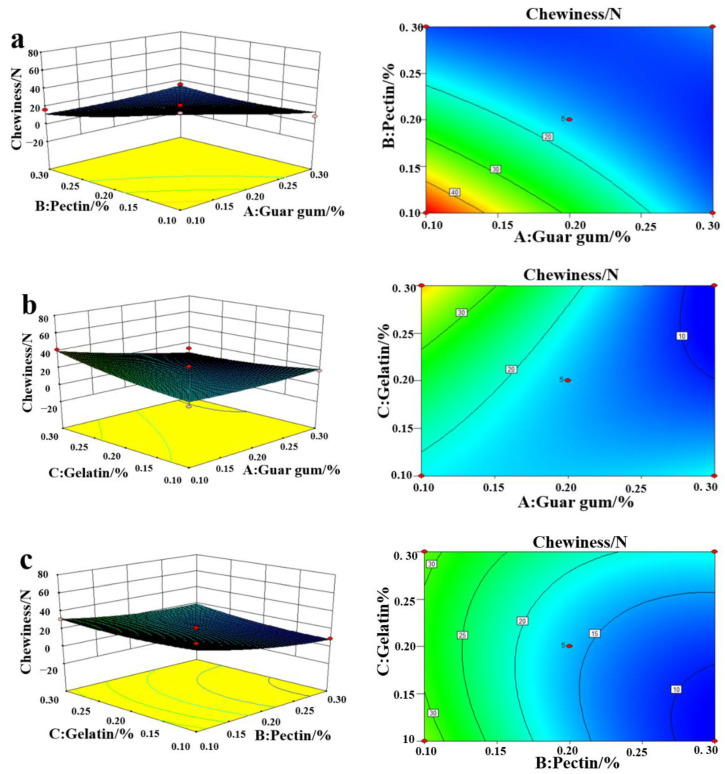
Response surface methodology of the interaction between guar gum and pectin (**a**), guar gum and gelatin (**b**), and pectin and gelatin (**c**) to chewiness.

Figure 1 shows the effect of the interaction of guar gum, pectin, and gelatin on chewiness. The gradient of the RSM curve reflects the effect of each factor on the response value of chewiness. Figure 1a shows that with the increase in guar gum and pectin concentration, the chewiness of the FRSB showed a decreasing trend. As shown in Figure 1b, with the increase in gelatin and guar gum concentration, the chewiness of the FRSB showed a slightly increasing trend. As the pectin concentration increased, the chewiness of the FRSB showed a decreasing trend, while the gelatin caused an increasing trend in chewiness, as illustrated in Figure 1c. The combination of pectin and cellulose creates a basic skeletal structure in the vacuum frozen product, providing some rigid support for the sample [24]. Gao et al. found that with the addition of the gelatin concentration, the chewiness of the product increased. Therefore, the chewiness of the product increased accordingly after the compounding of the three edible gums in order to produce better texture properties [25].

### 2.3. Influence of the Amount of Edible Gum Added on the Hardness Evaluation

**Figure 2:**
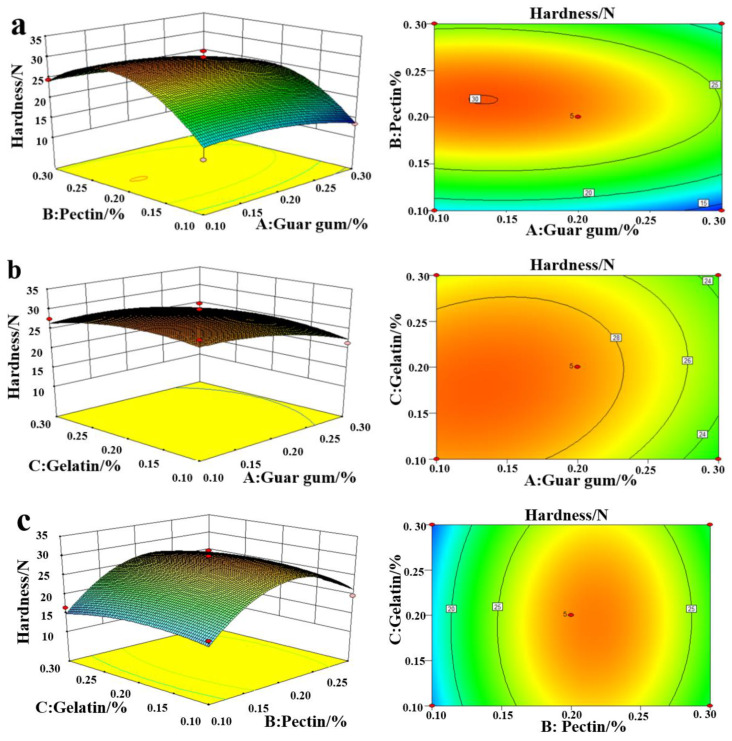
Response surface methodology of the interaction between guar gum and pectin (**a**), guar gum and gelatin (**b**), and pectin and gelatin (**c**) to hardness.

Figure 2 shows a 3D map of the RSM with the hardness evaluation as the response value. As shown in Figure 2a, the hardness first increased and then decreased with the pectin and guar gum concentration; Figure 2b shows that the hardness index decreased with the increase in guar gum and gelatin content; as illustrated in Figure 2c, the hardness index first increased and then decreased with the increase in pectin and gelatin. Du et al. studied that with the addition of pectin, the hardness and crispness of vacuum freeze-dried restructured apples improved, respectively [26]. Compounding edible gum can enhance the hardness of the product. Marfil et al. showed that the three-dimensional network structure of the modified starch compounded with gelatin had microporous structures with pore sizes of 10–50 μm. The denser the network structure, the higher the hardness [27]. The possible reason for this is that with the increase in concentration, the edible gum solution penetrates the tissue, leading to a higher porosity and forming a dense network microstructure, resulting in an increase in FRSB hardness [28,29,30].

### 2.4. Influence of the Amount of Edible Gum Added on the Organoleptic Evaluation

**Figure 3:**
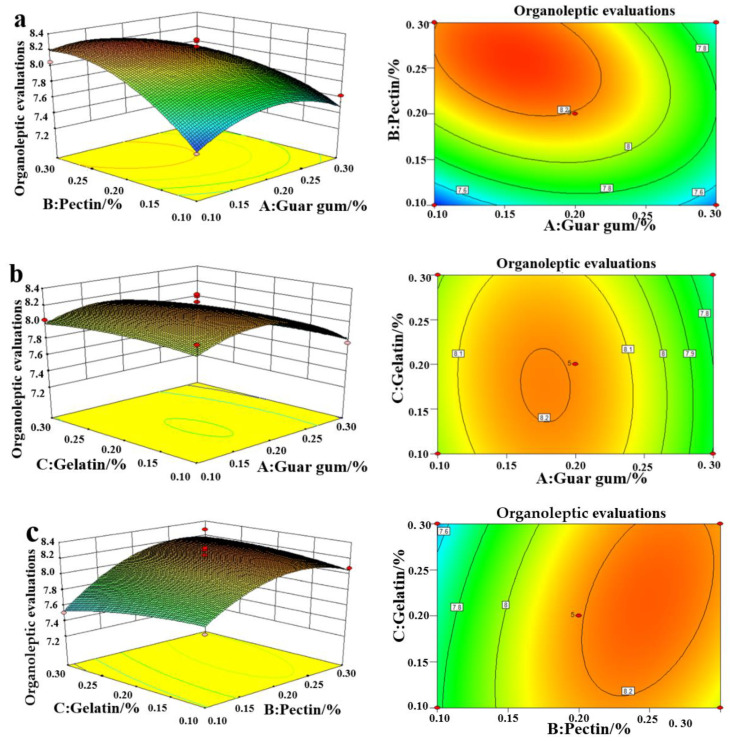
Response surface methodology of the interaction between guar gum and pectin (**a**), guar gum and gelatin (**b**), and pectin and gelatin (**c**) to organoleptic evaluations.

Figure 3 shows the organoleptic response value and uses the 3D value of the RSM to analyze the influence of the interaction between guar gum, pectin, and gelatin. It can be seen from Figure 3a that with the increase in the guar gum and the pectin concentration, the organoleptic score first becomes increased and then decreased. As shown in Figure 3b, with an increase in guar gum, the organoleptic score becomes first increased and then decreased and the organoleptic score becomes larger with an increase in gelatin concentration. With the increase in pectin concentration, the organoleptic score first increased and then decreased, and with the increasing gelatin concentration, the organoleptic score became slightly smaller. A plausible reason for this phenomenon is that the addition of edible gum changes the structure of FRSB, increasing its porosity and making it crispier. Therefore, there is a change in organoleptic evaluation [31].

### 2.5. The Optimal Combination Addition and Verification

Using Design Expert 8.0 software to determine the optimal combination addition, the FRSB was obtained by adding 0.1% guar gum, 0.22% pectin, and 0.30% gelatin. Experiments were carried out according to the optimal conditions obtained by the software, and three parallel experiments were performed for each group in order to obtain the optimal levels of chewiness, hardness, and organoleptic evaluation of the dried samples. The predicted values of chewiness, hardness, and organoleptic evaluations by the quadratic regression models mentioned above were 36.35, 26.82, and 8.09, respectively. The experimental values were 36.20 ± 0.80, 26.47 ± 0.95, and 8.10 ± 0.10, respectively. The results demonstrated that there was no obvious difference between the predicted and experimental values. Therefore, the optimal combination parameters of edible gum added by RSM optimization, namely chewiness, hardness, and organoleptic regression model prediction, are reliable.

### 2.6. Comparison of the Untreated Samples and the Samples Treated with the Optimal Combination Addition

#### 2.6.1. Moisture Content, Yield, Chewiness, Hardness, the Total Color Difference, Organoleptic Evaluations, and Pore Structure Analysis

Table 2 shows a comparison of the moisture content, yield, chewiness, hardness, the total color difference (Δ*E*), organoleptic evaluations, and pore structure analysis between the untreated samples and the samples treated with the optimal combination addition. The moisture content of the FRSB with the addition of edible gum was 0.8% higher than that of the untreated samples. This may be because the added edible gums could enhance the water-binding ability and moisturizing effect, delaying the formation of ice crystals during freezing and the dehydration action during drying, which contributed to a higher moisture content in the dried samples with the optimal combination addition of the three edible gums [32,33]. The yield of the FRSB in the treatment group with the optimal combination (0.10% guar gum + 0.22% pectin + 0.30% gelatin) and the FRSB in the control group without edible gum showed no significant difference (*p* > 0.05). Compared with untreated samples, the chewiness and hardness of FRSB in the treatment group with the optimal combination of edible gum additions increased by 82.04% and 27.09%, respectively. This may be because the edible gums and polysaccharide or cellulose were cross-linked during the drying process. Thereafter, the FRSB formed a tighter porous network microstructure, resulting in a harder product [28,29,30]. The Δ*E* of the FRSB of the treatment group was less than that of the control group without the addition of edible gum, indicating that the addition of edible gum could better maintain the original color of the product. The results show that the organoleptic level of the FRSB with the addition of edible gum was significantly higher than that without the addition of edible gum. This may be because edible gum endowed the FRSB with certain textural properties, and improved the index of brittleness in the organoleptic evaluation and the color of the product, which is crucial to the consumer’s acceptable level. Thus, the organoleptic evaluation of the product with the addition of edible gum was significantly higher than that of the sample without edible gum.

Pore structure indicators such as porosity, apparent density, pore area, and pore size distribution were used to quantify the microstructure of the dry products. This information is important for product design, selection of processing conditions, product organoleptic and textural properties, and shelf-life stability effects [34]. As shown in Table 2, the FRSB in the control group without edible gum had a lower porosity (29.33%) than the treatment group (32.34%). The total pore volume, average pore diameter, apparent density, and total pore area of the treatment group were larger than those of the FRSB without the addition of edible gum, which indicates that the addition of edible gum supports the overall structure of the FRSB, thereby increasing the pore size inside the block [35,36].

#### 2.6.2. Volatile Compounds Analysis

**Figure 4:**
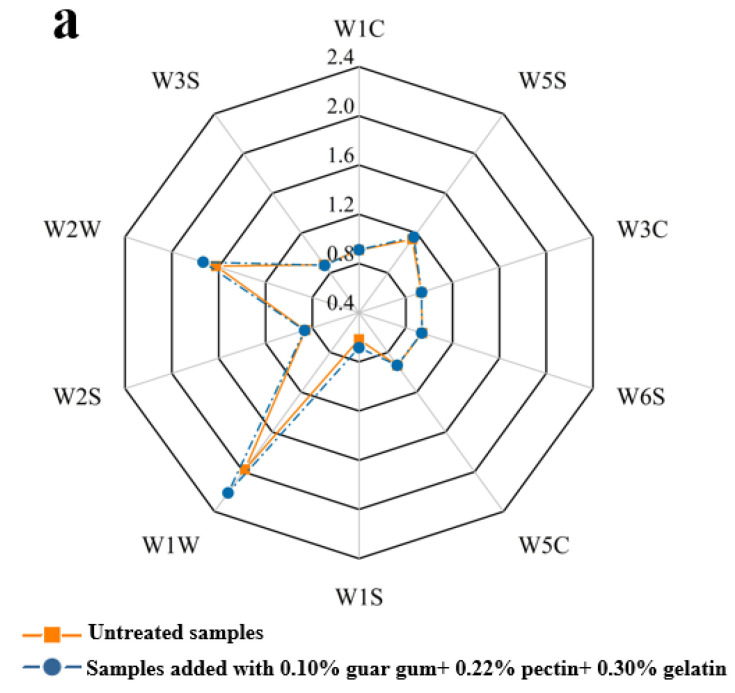

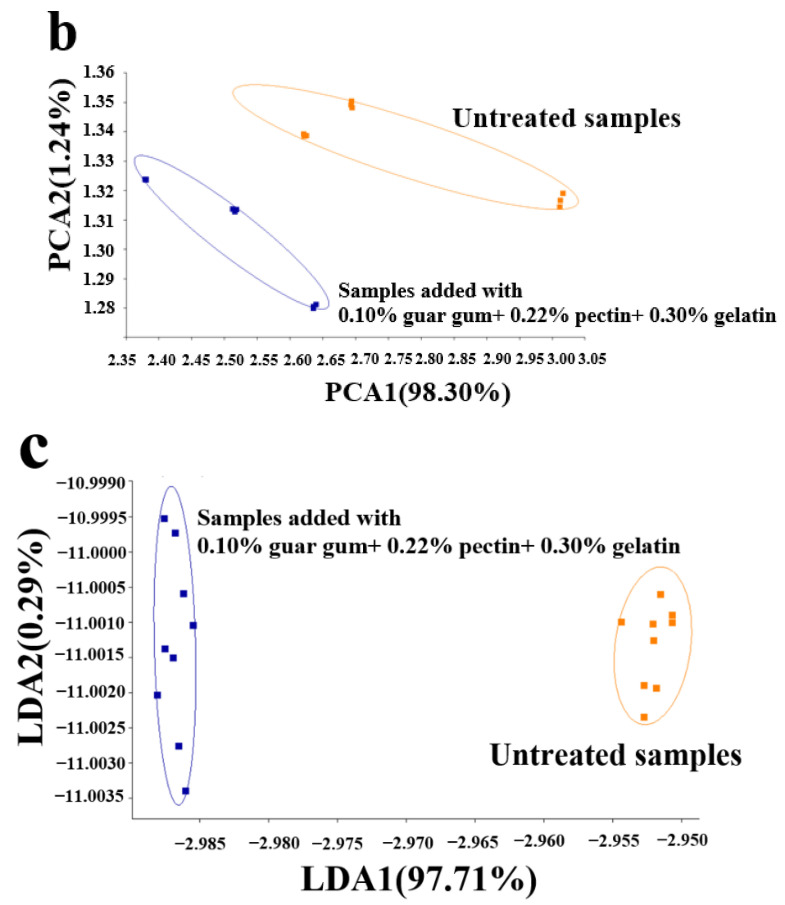
The radar map (**a**), principal component analysis (**b**), and linear discriminant analysis (**c**) of volatile compounds of untreated and treatment samples of the freeze-dried restructured strawberry blocks. Note: “W1C”means “aromatic, benzene”; “W3S” means “sensitive to long-chain alkanes”; “W2W” means “aromatic”; ”W2S” means “sensitive to alcohols, aldehydes and ketones”; “W1W” means “sensitive to sulfides”; ”W1S” means “sensitive to methyl compounds”; “W5C” means “broad range, sensitive to nitrogen oxides”; “W6S” means “mainly selective to hydrides”; “W3C” means “sensitive to aromatic and ammonia”; “W5S” means “aromatic components of short-chain alkanes”; “PCA” means principal component analysis; “LDA” means linear discriminant analysis.

The electronic nose is a new type of analytical instrument that imitates the human taste mechanism. Multiple taste sensors respond differently to different components in the sample, to be assessed in order to achieve a quantitative analysis of the overall taste characteristics. The radar chart clearly reflects the effect of the addition of edible gum on the response of FRSB to different sensors. As shown in Figure 4a, compared with the untreated groups, the response values W1W and W2W of FRSB with added edible gum were more. This could be explained by the selective diffusion theory and the microregion entrapment theory. With the progress of dehydration in the freeze-drying process, the edible gums could cross-link with some substances in the sample tissue, forming various microregions, which acted as physical barriers to entrap the volatile compounds, reducing the diffusion permeability of the volatiles, and the mobility of volatile compounds decreased, which could account for the higher content of the volatile compounds in the FRSB added with edible gums [37,38,39]. 

Figure 4b,c shows the comparison of principal component analysis (PCA) and linear discriminant analysis (LDA) of the FRSB in the treatment group with the optimal combination of edible gum added and the control group without edible gum. The results of PCA and LDA are consistent with the radar map results, demonstrating that the profiles of volatile compounds in the two groups presented an obvious difference. Figure 4b shows that the sum of the contribution rates of the two PCAs in the FRSB was higher than 90.00%, so the images drawn by PC1 and PC2 could express the main information characteristics of the FRSB well. Overall, in the PCA plot, volatile flavor compounds could be clearly distinguished between the two samples. Figure 4c shows that the sum of the contribution rates of the two LDAs of the FRSB in the treatment group and the control group without edible gum was higher than 90.00%, so it could also express the main information characteristics of the FRSB. In the LDA plot, volatile flavor compounds could be clearly distinguished between the two samples.

#### 2.6.3. Microstructural Analysis

**Figure 5:**
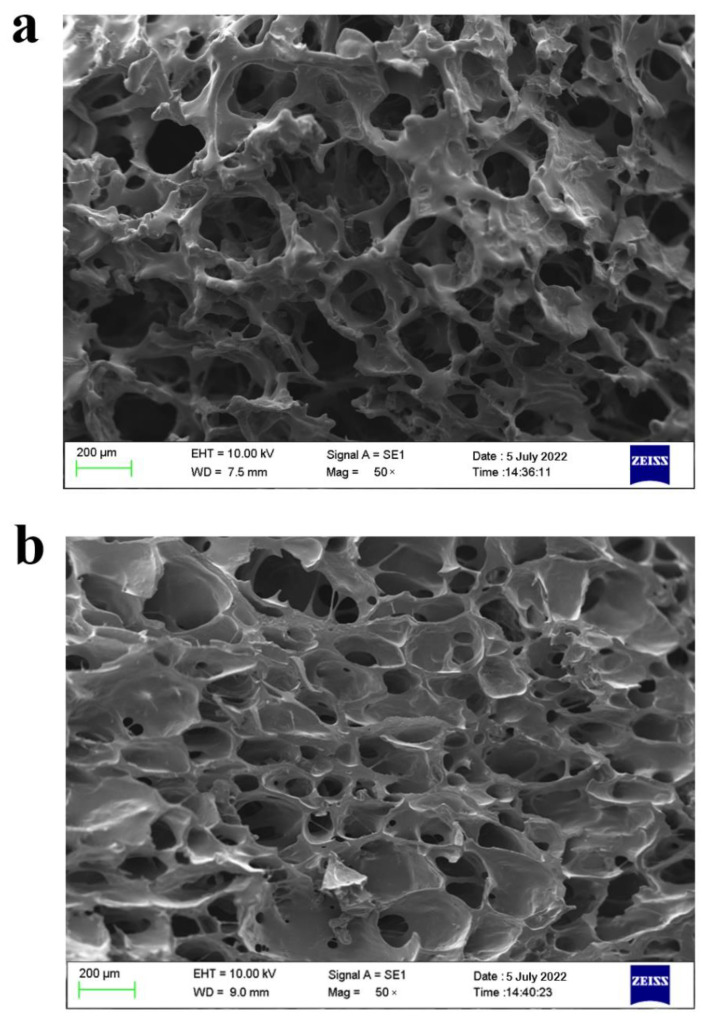
Scanning electron microscopy analysis of the freeze-dried restructured strawberry blocks (magnification 50×): (**a**): untreated samples, (**b**) samples with the optimal combination of edible gum additions.

Figure 5 shows the microstructure of the FRSB in the optimal combination of edible gum in the treatment group and the control group without edible gum. Figure 5a shows the microstructure of the untreated sample, which has a loose overall structure and irregular pore structure of different sizes. Figure 5b shows that the FRSB with the optimal combination of edible gum additions has a dense porous structure, which may be due to the thickening and coagulation effect of the edible gum. This pore structure promotes the high porosity of the FRSB. At the same time, it shows improved textural characteristics, such as chewiness and hardness [40]. 

## 3. Materials and Methods

**Figure 6:**
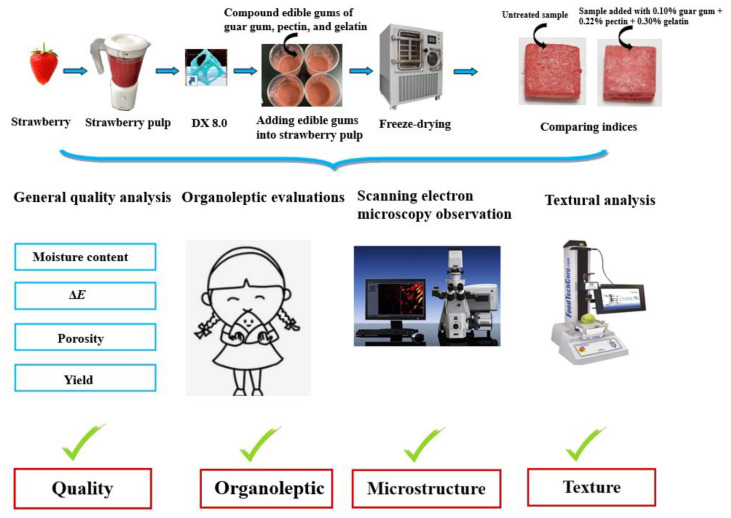
Schematic overview of the experimental procedure.

A schematic overview of the experimental procedure is illustrated in Figure 6.

### 3.1. Materials

Fresh strawberries (*Fragaria × ananassa “Red Face”*) were purchased from Lai-yang Haitel Food Co.; Ltd.; Qingdao, China. The strawberries were planted in the local greenhouse in September 2021 and harvested in March 2022. Sucrose, maltodextrin, and sodium caseinate were purchased from Henan Wan-bang Chemical Technology Co.; Ltd.; Zhengzhou, China.

### 3.2. The Preparation of Freeze-Dried Restructured Strawberry Blocks

Based on the results of the pre-experiments, fundamental additives, including 9% sucrose, 2% maltodextrin, and 1% sodium caseinate (mass/mass, according to the mass of the raw materials of strawberry), were added in the strawberry pulp in order to achieve a good quality of dried products. The strawberries were crushed three times with a crusher (JYZ-D51, Shandong Jiuyang Industrial Co.; Ltd.; Jinan, China), each time for 40s. Then, 9% sucrose, 2% maltodextrin, and 1% sodium caseinate were sequentially added into the strawberry pulp while stirring well. For the addition of the three edible gums, 17 treatment group experiments were designed according to the method of Box–Behnken in RSM. Different concentrations of pectin, gelatin, and guar gum were added sequentially and mixed well. The mixture was homogenized for 90 s at a speed of 65,000 r/min using a homogenizer (FJ200-SH, Shanghai Huxi Industrial Co.; Ltd.; Shanghai, China). 

The above prepared strawberry pulp was placed into a 2 cm × 2 cm × 1 cm stainless steel mold and placed in vacuum freeze-drying equipment (SCIENTZ-50F, Ningbo Scientz Biotechnology Co.; Ltd.; Ningbo, China). During the vacuum freeze-drying process, the strawberry material was frozen for 4 h, and the vacuum pump was then started when the center temperature of the sample fell below −40 °C. When the vacuum was below 100 Pa, the plate heating program was initiated. The heating temperature–duration times were as follows: −30 °C-2 h, −20 °C-2 h, −10 °C-2 h, 0 °C-2 h, 10 °C-2 h, 20 °C-2 h, 30 °C-2 h, 40 °C-2 h, and 50 °C-4 h. When the vacuum freeze-drying process was finished, the resultant FRSB was demolded, packed into an aluminum foil bag with a desiccant, refrigerated, and stored at −18 °C for future use.

### 3.3. Response Surface Methodology for the Optimization of Compound Edible Gums Addition

In this test, the addition of guar gum (A), pectin (B), and gelatin (C) was subjected to a three-factor three-level RSM analysis test, and the chewiness, hardness, and organoleptic indices were used as the response indicators. Design Expert 8.0 software was then used to conduct the Box–Behnken design with 17 groups of experimental programs. The experimental design and results are presented in Table 3. Thereafter, the optimal combination addition of the three edible gums was obtained and verified by experiments again, in which the group without edible gum addition was used as the control to compare the indices or properties of the dried samples such as the moisture content, yield, chewiness, hardness, total color difference, organoleptic evaluation, volatile compounds, porosity, and microstructure.

### 3.4. Determination of Moisture Content

The moisture content was determined by oven drying according to the method of Aktar and Adal, with slight modification [41]. 

About 2.0–3.0 g of the dried sample was dried in an oven at 105 °C until a constant weight was reached, and the weight loss was used to calculate the moisture content of the sample. All of the experiments were performed in triplicate.

### 3.5. Determination of Yield

Before the strawberry material was put into the mold and its net weight was measured, and after vacuum freeze drying, the FRSB was weighed. The weight before freeze-drying and the weight percentage after freeze-drying was the yield. This was calculated using Equation (4). Each group of samples was measured three times, and the results were averaged.

(4) $$y\;=\frac{x_{1}}{x_{2}}\;\times \;100$$

where *y* is yield (%),$\;x_{1}$ is the mass of the FRSB before freeze-drying (g), and $x_{2}$ is the mass of the FRSB after freeze-drying.

### 3.6. Determination of Texture Profile Analysis

The TPA of the dried sample was determined by a texture analyzer (TMS-PRO, Food Technology Corporation, Sterling, VA, USA). The “TPA-1000 N” test program was selected, which uses a test probe that is 50 mm in diameter, with the test parameters set as follows: initial force of 0.5 N, height of 20 mm, deformation of 30%, and detection speed of 60 mm/min. The samples were mechanically compressed twice to obtain the relevant texture parameters, and the parameter of chewiness was used. Each group of samples was analyzed three times, and the results were averaged.

### 3.7. Determination of Puncture

The samples were punctured using a texture analyzer (TMS-PRO, Food Technology Corporation, Sterling, VA, USA), and the “puncture-1000 N” test program was selected. The parameters were set as follows: initial force of 0.5 N, puncture distance of 2 mm, return distance of 20 mm, and experimental test speed of 60 mm/min. The hardness of the puncture test serves as another texture parameter. Each group of samples was analyzed three times, and the results were averaged.

### 3.8. Determination of Total Color Difference

The color of the FRSB was measured using a colorimeter (NH310, Shenzhen 3nh Technology Co.; Ltd.; Shenzhen, China). Before using the machine, it was calibrated, and each sample was measured in triplicate. For the initial strawberry pulp, *L*_0_^∗^ = 55.43, *a*_0_^∗^ = 10.99, *b*_0_^∗^ = 7.39, Δ*E* was calculated using Equation (5). Each group of samples was analyzed three times, and the results were averaged.

(5) $$\Delta E=\sqrt{{\left(L^{*}-{L_{0}}^{*}\right)}^{2}+{\left(a^{*}-{a_{0}}^{*}\right)}^{2}+{\left(b^{*}-{b_{0}}^{*}\right)}^{2}}$$

where Δ*E* is the total color difference of the sample; *L*^∗^, *a*^∗^, and *b*^∗^ are the color readings of lightness, red, and yellow, respectively, of the dried samples, and *L*_0_^∗^, *a*_0_^∗^, and *b*_0_^∗^ are the average color readings of the fresh strawberry pulp samples.

### 3.9. Determination of Organoleptic Evaluation

A group of 10 food students with the ability to identify organoleptic characteristics were invited as reviewers to conduct an analysis and evaluation after basic training in organoleptic evaluation. The samples were randomly provided to the organoleptic reviewers. Before the evaluation, their mouths were rinsed with water to evaluate the brittleness, stickiness, sweetness, and sourness of the samples according to a nine-point intensity scale, with “1” meaning the lowest, and “9” being the strongest. The strength of the sample indicators was expressed in order from the lowest to the highest, independent evaluation was required, and there was no communication between them.

### 3.10. Determination of Volatile Compounds

The volatile compounds in the samples were determined using an electronic nose (PEN3 electronic nose, Airsense, Germany). Each group of samples was placed in a 100 mL glass beaker, sealed with plastic wrap, and allowed to stand for 20 min so that the volatile flavor substances of the samples filled the entire beaker. Before using the electronic nose, the machine was started and preheated for 30 min, after which the sampling tube was inserted into the beaker and the volatile components were measured three times per sample. The E-nose test program parameters were as follows: sample preparation time, 5 s; measurement time, 75 s. The PEN3 electronic nose simulates the human body’s olfactory system, including 10 metal oxide sensors (sensor serial number 1–10). The sensor model names and performance descriptions for sensitive substances are listed in Table 4. Three parallel experiments were performed for each group of samples, and the results were averaged.

### 3.11. Determination of Microstructure

Scanning electron microscopy (SEM, EVO-LS10, Oberkochen, Germany) was used to observe the microstructure of the FRSB. The FRSB was quickly frozen with liquid nitrogen to obtain a cross-sectional sample, and the observation section of the FRSB was adhered to a sample holder with a carbon conductive adhesive. Typical apparent photographs of the cross-section of the FRSB were taken for the microstructure analysis.

### 3.12. Determination of Pore Structure

The pore structure properties of the FRSB were determined and analyzed using an AutoPore IV series automatic mercury porosimeter (AutoPore IV 9510; Mecromeritics Instrument Co.; Ltd.; Norcross, GA, USA). The FRSB was placed into the mercury porosimeter, and through the action of pressure in the instrument, the mercury in the mercury porosimeter entered the pores of the material. A measure of the pore size and porosity of the sample was obtained indirectly using the amount of mercury intrusion.

### 3.13. Statistical Analysis

In this experiment, Design Expert 8.0 was employed for the RSM optimization test and analysis. The ANOVA and Tukey HSD’s test of SPASS 22.0 (IBM, Chicago, IL, USA) software were used for data analysis with a significance level of *p* < 0.05; the electronic nose data weer analyzed by WinMuster software that comes with the electronic nose system; other data were analyzed and plotted by Origin 2021 (Origin Lab.; Northampton, MA, USA) software.

## 4. Conclusions

Freeze-dried restructured food is an emerging popular product. In order to enhance the quality of FRSB, edible gums of guar gum, pectin, and gelatin were added and the combination was optimized using response surface methodology. The results indicated that the optimal combination addition of three edible gums was 0.10% guar gum, 0.22% pectin, and 0.30% gelatin, which contributed to the highest comprehensive qualities of the dried product, including chewiness, hardness, and organoleptic evaluations. In addition, compared with the untreated samples, the optimal combination of edible gum additions enhanced the moisture content and the textural properties of chewiness and hardness of the FRSB, while maintaining a better appearance, forming a higher porous network microstructure of the FRSB, which are the desirable quality attributes of the product. The findings in current work indicate that adding edible gum can improve the quality of freeze-dried restructured fruits and vegetables, which is a feasible method for the expansion of fresh strawberry sales and the development of the strawberry pulp processing industry.

## Figures and Tables

**Table 1:** Analysis of variance of the regression models for chewiness, hardness, and organoleptic evaluations.

Source	Chewiness				Hardness			Organoleptic Evaluations		
	DF	Sum of Squares	*F*-Value	*p*-Value	Sum of Squares	*F*-Value	*p*-Value	Sum of Squares	*F*-Value	*p*-Value
Model	9	1986.64	5.51	0.0174 *	481.51	6.66	0.0102 *	1.34	5.19	0.0205 *
A	1	518.90	12.95	0.0087 *	44.27	5.52	0.0512	0.12	4.19	0.0799
B	1	659.75	16.47	0.0048 *	68.91	8.58	0.0220 *	0.43	14.92	0.0062 *
C	1	48.22	1.20	0.3089	0.94	0.12	0.7425	0.0066	0.23	0.6457
AB	1	334.34	8.35	0.0233 *	0.90	0.11	0.7472	0.12	4.03	0.0846
AC	1	261.79	6.54	0.0377 *	2.37	0.30	0.6036	0.0016	0.056	0.8200
BC	1	34.81	0.87	0.3823	0.29	0.036	0.8543	0.034	1.19	0.3107
A^2^	1	16.39	0.41	0.5428	14.43	1.80	0.2218	0.31	10.69	0.0137 *
B^2^	1	61.69	1.54	0.2546	314.79	39.21	0.0004 *	0.27	9.35	0.0184 *
C^2^	1	38.28	0.96	0.3608	15.55	1.94	0.2067	0.011	0.40	0.5467
Residual	7	280.39			56.19			0.20		
Lack of fit	3	195.84	3.09	0.1523	31.24	1.67	0.3095	0.095	1.20	0.4171
Pure error	4	84.55			24.96			0.11		
Cor total	16	2267.04			537.71			1.54		

Note: “DF” means degree of freedom; “*F*-value” means the statistics of the F-test; “*p*-value” means significance; “*” means significant value was less than 0.05 (*p* <0.05). “Model” means the quadratic regression equation for chewiness, hardness, and organoleptic evaluation; “A” means the concentration of guar gum; “B” means the concentration of pectin; “C” means the concentration of gelatin; “AB, AC, BC” mean the interaction between the concentration of guar gum and pectin, guar gum and gelatin, pectin, and gelatin, respectively; “A^2^, B^2^, C^2^” mean the square of the concentration of guar gum, pectin, and gelatin, respectively.

**Table 2:** Comparison of indices for the untreated and treatment samples of the freeze-dried restructured strawberry block.

Index	Untreated Samples	Samples Added with 0.10% Guar Gum + 0.22% Pectin + 0.30% Gelatin
Moisture content (%)	4.00 ± 0.00 ^a^	4.80 ± 0.00 ^b^
Yield (%)	19.00 ± 0.12 ^a^	19.00 ± 0.15 ^a^
Chewiness (N)	6.50 ± 0.32 ^b^	36.20 ± 0.80 ^a^
Hardness (N)	19.30 ± 0.87 ^b^	26.47 ± 0.95 ^a^
Δ*E*	15.67 ± 0.22 ^a^	12.21 ± 0.89 ^b^
Organoleptic evaluations	7.03 ± 0.12 ^b^	8.10 ± 0.10 ^a^
Total pore volume (mL/g)	0.76 ± 0.05 ^b^	0.80 ± 0.07 ^a^
Average pore diameter (nm)	118.90 ± 1.16 ^b^	125.10 ± 0.93 ^a^
Apparent density (g/mL)	0.54 ± 0.04 ^b^	0.62 ± 0.03 ^a^
Total pore area (m^2^/g)	24.83 ± 0.04 ^b^	25.65 ± 0.12 ^a^
Porosity (%)	29.33 ± 0.25 ^b^	32.34 ± 0.74 ^a^

Note: Values are means ± SD of three repetitions; Data points in the same row with different letters (a and b) are significantly different (*p* < 0.05). “Δ*E*” means the total color difference.

**Table 3:** Experimental design of Box–Behnken in the response surface methodology.

Treatment Group	Guar Gum/%	Pectin/%	Gelatin/%	Chewiness/N	Hardness/N	Organoleptic Evaluations
1	0.20	0.30	0.30	12.92 ± 0.75	19.95 ± 0.62	8.28 ± 0.05
2	0.10	0.30	0.20	16.73 ± 0.54	24.46 ± 0.76	8.05 ± 0.03
3	0.20	0.20	0.20	21.65 ± 0.83	28.87 ± 0.50	8.33 ± 0.02
4	0.20	0.20	0.20	14.26 ± 0.56	29.97 ± 0.48	7.93 ± 0.11
5	0.10	0.20	0.30	41.25 ± 0.87	27.52 ± 0.54	8.03 ± 0.05
6	0.20	0.10	0.30	31.15 ± 0.82	16.54 ± 0.68	7.51 ± 0.03
7	0.30	0.20	0.30	12.85 ± 0.73	21.81 ± 0.43	7.58 ± 0.01
8	0.20	0.10	0.10	8.98 ± 0.52	19.72 ± 0.60	8.08 ± 0.01
9	0.10	0.20	0.10	13.29 ± 0.73	30.12 ± 0.80	8.12 ± 0.03
10	0.30	0.10	0.20	8.93 ± 0.49	13.43 ± 0.24	7.63 ± 0.02
11	0.20	0.20	0.20	12.18 ± 0.62	31.42 ± 0.78	8.24 ± 0.04
12	0.20	0.20	0.20	12.21 ± 0.60	24.81 ± 0.92	8.15 ± 0.02
13	0.30	0.20	0.10	17.25 ± 0.89	21.33 ± 0.34	7.75 ± 0.04
14	0.20	0.10	0.10	39.01 ± 0.76	17.39 ± 0.28	7.68 ± 0.04
15	0.20	0.20	0.20	20.56 ± 0.24	29.77 ± 0.76	8.31 ± 0.01
16	0.30	0.30	0.20	15.02 ± 0.65	21.35 ± 0.87	7.63 ± 0.05
17	0.10	0.10	0.20	47.21 ± 0.78	14.64 ± 0.46	7.37 ± 0.02

Note: Values are means ± SD of three repetitions.

**Table 4:** Sensors used and their main application in the PEN3 system.

Sensor Serial Number	Sensor Model Name	Performance Description for Sensitive Substances
1	W1C	Aromatic, benzene
2	W3S	Sensitive to long-chain alkanes
3	W2W	Aromatic
4	W2S	Sensitive to alcohols, aldehydes and ketones
5	W1W	Sensitive to sulfides
6	W1S	Sensitive to methyl compounds
7	W5C	Broad range, sensitive to nitrogen oxides
8	W6S	Mainly selective to hydrides
9	W3C	Sensitive to aromatic and ammonia
10	W5S	Aromatic components of short-chain alkanes

## Data Availability

Data is contained within the article.
